# Flexible and Efficient Security Framework for Many-to-Many Communication in a Publish/Subscribe Architecture

**DOI:** 10.3390/s22197391

**Published:** 2022-09-28

**Authors:** Roald Van Glabbeek, Diana Deac, Thomas Perale, Kris Steenhaut, An Braeken

**Affiliations:** 1 Department of Engineering Technology (INDI), Vrije Universiteit Brussel, Pleinlaan 2, B-1050 Brussels, Belgium; 2 Department of Electronics and Informatics (ETRO), Vrije Universiteit Brussel, Pleinlaan 2, B-1050 Brussels, Belgium; 3 Communications Department, Technical University of Cluj-Napoca, 400114 Cluj-Napoca, Romania

**Keywords:** IoT, WSN, MQTT, security, many-to-many, publish/subscribe, TLS

## Abstract

Message Queuing Telemetry Transport (MQTT) is a lightweight publish/subscribe protocol, which is currently one of the most popular application protocols in Internet of Things (IoT) thanks to its simplicity in use and its scalability. The secured version, MQTTS, which combines MQTT with the Transport Layer Security (TLS) protocol, has several shortcomings. It only offers one-to-one security, supports a limited number of security features and has high computation and communication costs. In this paper, we propose a flexible and lightweight security solution to be integrated in MQTT, addressing many-to-many communication, which reduces the communication overhead by 80% and the computational overhead by 40% for the setup of a secure connection on the client side.

## 1. Introduction

Wireless Sensor Networks (WSNs), which represent an important subset of the Internet of Things (IoT), are nowadays increasingly popular. They are used in a variety of domains and applications, such as smart home, smart health, smart manufacturing, environmental monitoring, etc.

One of the most popular and mature application protocols in IoT is the Message Queuing Telemetry Transport (MQTT) protocol. It is based on a highly decoupled publisher and subscriber model, in which the senders and receivers do not communicate directly, but via a central server.

In MQTT, senders are called publishers, receivers are called subscribers, and the central server is called the broker. The publishers define upfront the type of category or topic in which they will send information, the subscribers express their interest in the topics, and the broker takes care of the dissemination from publisher to subscriber based on the selected interests.

For the security in MQTT, authentication is of utmost importance. In order to trust the information coming from publishers, subscribers should be able to verify its origin. The same holds for a publisher in case a query is received from the subscriber.

Confidentiality can also be interesting, but is not always required, for instance in case of a simple outdoor temperature sensor. Other security features, such as non-repudiation, anonymity, and unlinkability, can play an important role in some situations. Consider the example of a smart home, in which an attacker should not be able to follow the communication pattern of the alarm sensor.

MQTTS represents the secured version of MQTT, relying on the standard Transport Layer Security (TLS) protocol. However, there are several problems with this approach. First, TLS only offers a limited set of security features: confidentiality and authentication from publishing device to broker and from broker to subscriber. Consequently, all messages are stored unencrypted and in case of a dishonest broker (e.g., a hacked broker), all messages can be retrieved. Next, anonymity is not possible with standard TLS. Moreover, as shown in [1,2], TLS still has very high demands with respect to communication and computational costs. Moreover, TLS communication is limited to one-to-one communication.

To overcome the above mentioned shortcomings, we have proposed a solution, resulting in the following main contributions of the paper.

- We have developed a global framework, offering three types of lightweight security options in a highly efficient way. In the highest security option, security features of authentication, confidentiality, non-repudiation, and anonymity are included.
- We have embedded this security framework into the context of MQTT communication, enabling secure many-to-many communication, while taking into account the trade-off between security and efficiency.
- We have implemented our proposed solution in a testbed and, compared with MQTTS, show that the communication overhead is reduced by 80% and the computational overhead by 40% for the setup of a secure connection on the client side.

## 2. Related Work

There exists in literature different approaches to integrate secure group key protocols for multicast communication. In [3], the use of a network multicast manager is proposed, which takes the role of administration of the different multicast groups among the nodes in a wireless sensor network, participating in the communication. The authors proposed a symmetric-key-based protocol, which enables to achieve confidentiality, integrity, and mutual authentication among the members of the group. In [4], a robot-assisted network bootstrapping technique is proposed to support various multicast group semantics. The authors established a location-class-aware symmetric key management framework offering confidentiality and authentication protection. Another type of architecture in which group key communication is proposed is in the context of fog computing. In [5], a group key between the fog device and the edge devices is constructed based on partial secrets shared by the server with each of the IoT devices belonging to that group. Their scheme relied on elliptic curve cryptography and Lagrange interpolation. In the schemes above, the participants can individually derive the group key at any moment. This is different from secret sharing schemes, where a so-called dealer distributes the shares to the different participants in such a way that only authorized subsets of participants can reconstruct the secret when they are all together. A recent survey on Perfectly Secure Verifiable Secret-sharing is given in [6], where perfect means that computationally unbounded adversaries are tolerated. Using quantum cryptography, also a verifiable multi-dimensional (t,n) threshold secret sharing scheme has been proposed [7]. However, this type of schemes are not very practical for the generation of a group key, due to the strict requirement of the need to combine all secrets at the same time.

As the main focus of the paper is the proposal of a security framework to enable many-to-many communication with the MQTT protocol, we limit the rest of the related work on providing an overview of the security schemes for multicast communication protocols, added to the most well-known application protocols Constrained Application Protocol (COAP) and MQTT in literature.

A significant amount of research exists on multicast communication with the CoAP protocol. One of the first approaches was to maintain regular DTLS sessions between each pair of sender node and listener node [8]. However, this approach is not scalable and requires a very high amount of resources. In [9], the Axiom protocol was proposed in which unicast communication was secured by means of an individual key, derived from the group key. This approach clearly does not offer authentication as every member of the group is able to derive such a key. As an improvement, the authors proposed to rely on a trusted third party for the key management [10], but this approach was only valid for unicast DTLS communication.

In [11], a datagram TLS (DTLS)-based secure group communication scheme for IoT has been proposed, which outperforms previous related work [8,9,10]. Compared to [8,9], with the scheme proposed in [11] every sender in the group is able to send multicast messages, not only one per group. It is based on a centralised approach in which a group controller establishes unique keys among the different group members by means of DTLS. The sender sends multicast requests and the listener nodes reply with a unicast response. The main weakness of this scheme is that the security completely relies on the trustworthiness of the group controller.

In publish/subscribe architectures, which are the basis of MQTT, most of the proposals are limited to one-to-one communications. Peng et al. propose an identity (ID)-based publish/subscribe protocol in [12] to avoid the burden of classical public key infrastructure (PKI). However, due to the inherent nature of ID-based cryptography, the system is vulnerable to key escrow attacks. Moreover, the proposed approach is still limited to one-to-one communication.

In [13], a proxy re-encryption (PRE) scheme is integrated in the publish/subscribe architecture. In a PRE scheme, a publisher encrypts information using a public key without knowing the subscribers. Then, a re-encryption key is generated without interaction between publishers and subscribers to convert the encrypted data of the publisher such that it can be decrypted by the intended receiver. Polyakov et al. in [13] proposed two multi-hop unidirectional PRE schemes being secure for indistinguishability under chosen-plaintext attack (IND-CPA), relying on the Ring Learning with Error (RLWE) key switching approach from the lattice based homomorphic encryption literature. The main drawback of the scheme is that a fully trusted interactive policy authority needs to be present in the scheme. Moreover, the scheme is also limited to one-to-one communication and possesses a significant computational and communication cost due to the underlying cryptographic algorithms.

In [1,2], a resource efficient end-to-end publish/subscribe based security scheme relying on elliptic curve operations has been proposed and compared with the traditional TLS-based scheme. However, the proposed schemes still contain a significant number of handshakes and a significant communication cost. Moreover, both schemes do not involve group-based communication as the broker needs to develop a separate message for each subscriber of the topic. In the scheme of [1], the broker is considered as a fog device, but needs to be completely trusted as a common pre-shared key between device and fog is required. The scheme of [2] relies on a honest but curious broker for the confidentiality, but not for the authentication.

Additionally, the scheme of [14] is developed for fog computing architectures where the fog takes over the role of broker. It is again a one-to-one communication scheme proposing the use of the TLS-ECDHE-PSK mode and, thus, requiring the existence of a preshared key for authentication purposes. Their proposal is compared against different cipher suites of TLS like ECDHE-RSA, ECDHE-ECDSA, and TLS-PSK.

Attribute-based encryption (ABE) schemes enable end-to-end encryption and access control only for subscribed clients to decrypt the publisher’s message and, thus, are inherently many-to-many. Several ABE-based proposals, applied to the publish/subscribe architecture, exist in literature. In [15], Ion et al. propose a scheme which combines ABE, multi-user encrypted search, and PRE. The main goal of the scheme is to avoid that a broker learns something about the content, event, or filtering constraints of the subscribers, without the need to share keys between publishers and subscribers. The keys of all participants are all generated by a trusted third party. Due to the usage of computationally intensive pairing operations, the scheme is not feasible for IoT applications with constrained devices.

Tariq et al. [16] propose a brokerless publish/subscribe system with the aid of Ciphertext policy attribute-based encryption (CP-ABE), which embeds authorization policies into the ciphertexts and also relies on compute intensive pairing operations. A separated key is needed for each authorization credential by each publisher and subscriber, resulting in very complex key management on the side of the participants in the publish/subscribe protocol.

Duan et al. [17] propose a bidirectional policy matching scheme and a fully homomorphic encryption for the encryption of published events in order to enable one-to-many communication, providing both data and service privacy. A Certificate Authority (CA) derives both private and public keys for all entities. Again, this scheme requires too much computational effort for resource constrained devices.

A completely different approach to the topic is given in [18], where differential privacy is applied instead of encryption. By adding noise to the data, privacy of the measurements is obtained, causing a reduction in accurateness of the data.

In [19], Su et al. propose the MQTT Thing-to-Thing Security (MQTT-TTS) framework providing thing-to-thing security without data leaks. It consists of four different modes, symmetric encryption, asymmetric encryption, hash function, and user-defined mechanisms. No details are provided on key management. It is notable that it provides a method to include these modes, making it compliant with the original MQTT protocol.

For this work, we became inspired by the approach in [19], but we include a more coherent framework by adding proper key management and by enabling many-to-many communication. We avoid computationally intensive operations and huge key management burden typically present in attribute-based schemes. As a consequence, we do not offer privacy in service policies or relations among publishers and subscribers, but instead include device accountability in all our proposed modes, such that any receiver is able to verify the origin of the message. Moreover, the CA is not aware of the unique private key of each of the devices.

## 3. Background and Preliminaries

### 3.1. Architecture and Attack Model

**Figure 1:**
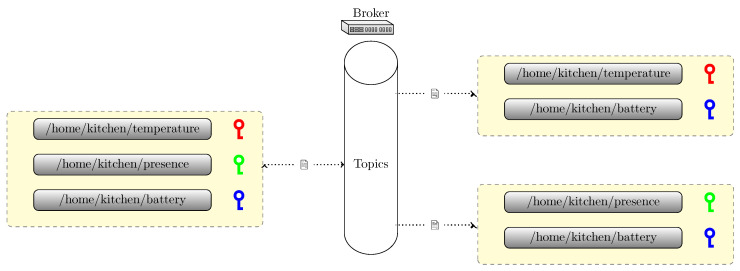
The broker in MQTT routes messages between groups of publishers and subscribers.

We consider a publish/subscribe setting, as shown in Figure 1. Different publishers are connected to different subscribers. Publishers send their data, which are forwarded by the broker to the interested subscribers. On the other hand, subscribers can also send a query via the broker to the group of publishers. This results in a many-to-many communication pattern.

An external party, called the CA, takes care of the registration and distribution of the key material and is considered to be fully trusted. The owner of the devices first makes sure that the devices, together with their topics of interest and topics on which they publish, are registered at the CA. Based on that, the CA starts the derivation of the required keys in the scheme. Each device has its unique private key and both groups of publishers and subscribers each have their own common private key.

The broker is considered honest but curious. It means that it will do all the required actions, but is curious in deriving the message for its own purposes, such as, for instance, selling the data. There are two options for storage at the broker side: either the storage is limited to the group public keys of the publishers and subscribers, or the broker stores all the public keys and, thus, in addition also the individual public keys of the devices.

We further assume that the attackers behave like those described in the Dolev-Yao security model [20], being able to execute both passive and active attacks. As a consequence, they do not only eavesdrop on the channel, but they can also actively manipulate by deleting, modifying, adding, etc., messages to the channel.

### 3.2. Security Features

In our proposed framework, we consider the following three options of combinations of security features.

- Option 1: Authentication and non repudiation. In this option, the receivers are able to verify the origin and content of the message. The sender is unable to afterwards deny the submitted message. This situation is typically applied in the case of environmental sensors, measuring temperature, humidity, etc.
- Option 2: Authentication, non repudiation and confidentiality. Here, nobody, not even the broker, except the subscribers are able to derive the message sent by the publishers. The broker is able to verify the authentication of the publisher in a non-repudiable manner. A typical use case for which this security level can be of interest are sensors measuring air quality and pollution. In order not to cause panic, it can be good to only let authorized people analyze the data.
- Option 3: Authentication, confidentiality, anonymity, unlinkability, and non-repudiation. Finally, in the full option, anonymity and unlinkability are also included. These features ensure that an attacker is unable to derive patterns in the communication of a set of sensors. For instance, sensors and actuators related to presence, door openers, alarms, and lamps should best keep their anonymity and unlinkability in order to avoid patterns by attackers to derive the real presence of the owner in the house. The same holds for sensors attached to a body sensor network of the patient in order to avoid tracking of a patient.

### 3.3. Cryptographic Operations

First, hash operations $H(M_{1}∥M_{2})$ on the concatenated message $M_{1}∥M_{2}$ are needed. Hash operations, such as SHA2 or SHA3, should be resistant against collision, pre-image, and second pre-image attacks.

Next, encryption operations relying on a symmetric key *K* and applied on the message *M* to obtain the ciphertext *C* will be used and are denoted by $E_{K}\left(M\right)$.

Finally, for the public key-based operations, we will rely on elliptic curve cryptography (ECC) [21], which offers more lightweight solutions than classical RSA. ECC is based on the algebraic structure of elliptic curves (ECs) over finite fields. We denote the curve in the finite field $F_{p}$ with *p* a large prime by $E_{p(a,b)}$, defined by the equation $y^{2}=x^{3}+ax+b$ with *a* and *b* two constants in $F_{p}$ and $\Delta =4a^{3}+27b^{2}\neq 0$. The base point generator of $E_{p(a,b)}$ of prime order *q* is denoted by *G*.

The EC multiplication $R=rP=(R_{x},R_{y})$ with $r\in F_{q}$ and $R_{x},R_{y}\in F_{p}$ results in a point of the EC. The security of ECC is based on the Elliptic Curve Discrete Logarithm Problem (ECDLP) and the Elliptic Curve Diffie–Hellman Problem (ECDHP) [21].

Three well established ECC-based algorithms are used, Elliptic Curve Integrated Encryption Scheme (ECIES), Schnorr signature, and Elliptic Curve Qu Vanstone (ECQV).

- Elliptic Curve Integrated Encryption Scheme [22]. ECIES enables efficient encryption to a particular receiver with public key $Q_{R}$ using a random EC point R=rG. The common secret key equals to $K=rQ_{R}$. Since $Q_{R}=d_{R}G$, the receiver is able to derive the same key $K=d_{R}R$ if *R* is added to the message.
- Schnorr signature [23]. Denote the key pair of the sender *S* by $\left(d_{S},Q_{S}\right)$ with $Q_{S}=d_{S}G$, where $d_{S}$ represents the private key and $Q_{S}$ the public key. To sign a message *M* with the Schnorr signature scheme, the sender first chooses a random value $v\in F_{q}^{*}$ and computes V=vG. Next, it derives h=H(M∥V), resulting in the actual signature $s=v-hd_{S}$. Using V,s, everybody knowing the public key of the sender $Q_{S}$ is able to verify the signature on *M* by checking the equality of the equation $sG=V-hQ_{S}$.
- Elliptic Curve Qu Vanstone certificates [24]. ECQV certificates are a lightweight alternatives of the classical X509 certificates and are typically used in IoT applications. In addition, they have interesting security features, as they do not need a secure channel between the certificate authority (CA) and the device requesting a certificate. In addition, they offer protection against key escrow attacks, as the CA is also not aware of the private key established by the device during the protocol. Assume the key pair of the CA is defined by $\left(d_{CA},Q_{CA}\right)$ and is publicly available and trusted.
  In the ECQV protocol, the device chooses a random value $r_{1}\in F_{q}^{*}$ and computes $R=r_{1}G$. This value, together with its identity and eventually a proof of identity, $R,ID_{1}$, are sent to the CA. The CA then also chooses a random value $r_{2}\in F_{q}^{*}$ and computes $R=r_{2}G$. The certificate $C_{1}$ is then defined as $C_{1}=R_{1}+R_{2}$. Next, the CA derives the auxiliary information $a_{1}=H\left(ID∥C_{1}\right)r_{2}+d_{CA}$ for the device to derive its private key.
  Based on the received data $a,C_{1}$ of the CA, the device is able to compute its key pair $\left(d_{1},Q_{1}\right)$. Here, $d_{1}=a_{1}+H\left(ID_{1}∥C_{1}\right)r_{1}$ and $Q_{1}=d_{1}G=H\left(ID_{1}∥C_{1}\right)C+Q_{CA}$. Only if the last equality is correct, the device accepts the key pair. Any other entity is able to derive the public key $Q_{1}$ knowing $ID_{1},C_{1}$ by using this last equality. Note that also an expiration time can be included in the hash of the computations.

### 3.4. MQTT

The MQTT protocol [25] is a simple machine-to-machine communication protocol that sits on top of the Transport Control Protocol (TCP) and is used to transport, both bandwidth and energy efficient, all types of sensor data gathered by a gateway to a server.

The MQTT protocol inherits all aspects of a publisher/subscriber model, as shown in Figure 1.

- Both publisher and subscriber are spatially separated. They only need to know the hostname/IP and port of the broker in order to send and receive messages.
- Decoupling in time is possible as the broker can store the messages for clients that are not online.
- The communication flow in MQTT works asynchronously. This means that there are no tasks blocks while awaiting or publishing a message.

In MQQT, the client has three options for the selection of level of Quality of Service (QoS), which is determined based on the network dependability and application logic. At the lowest level, QoS 0, the messages are delivered at most once and no delivery is guaranteed. In the QoS level 1, it is guaranteed that the message is delivered at least once to the receiver, while for the highest level, QoS 2, there is no message loss or duplication as messages are exactly once published.

For the proposed security process, in particular the key initialization and the key update phase, it is essential that the nodes receive the latest updates from the broker, since otherwise the node will fail to send and receive anything on the updated topic until a new node joins or leaves the topic. QoS in MQTT originally only applies to PUBLISH packets but could be extended to the key update packets to maintain the nodes with the correct keys. Since the security mechanisms in the protocol (e.g., use of timestamps) already offer protection against replay attacks, from a security point of view, QoS 1 is sufficient.

## 4. Framework

We distinguish four different phases in the actual operation, set-up phase, key initialization, secure communication, and update. Each of them will now be discussed.

### 4.1. Set-Up

Each device possesses a unique identity $ID_{i}$, based on the identity provided by the manufacturer. The action rules among the different devices are programmed by the owner. The public key of the CA is also pre-loaded on each of the devices, which is used to verify the authenticity of the messages received from the CA. In addition, the owner registers the topics corresponding to the devices for the publishing and subscribing processes.

### 4.2. Key Initialization

**Figure 2:**
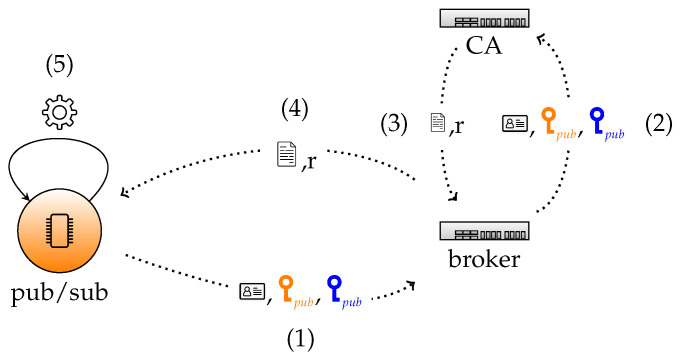
Certificate request and reception between a client node and a broker communicating with a certificate authority. (1) ECQV request of device, (2) ECQV request of client forwarded by broker to CA, (3) ECQV response of CA, (4) ECQV response forwarded by broker to device, and (5) reception and verification of device.

**Figure 3:**
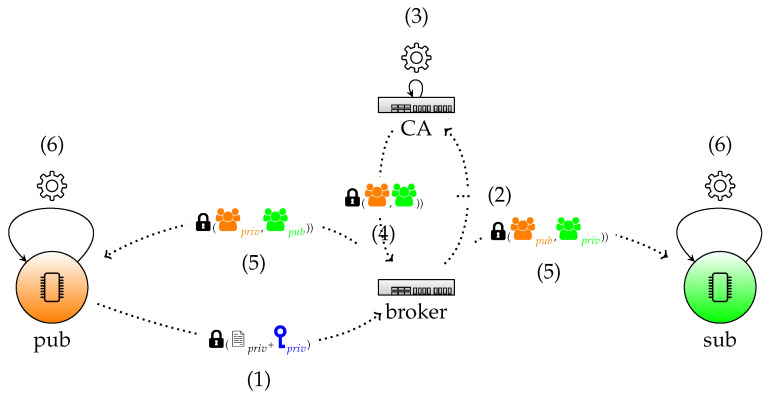
Confirmation sending and group key reception. (1) Group key request of client, (2) group key request of client forwarded by broker to CA, (3) construction of group-key-related info by CA, (4) response of CA to broker, (5) response of broker to both publishers and subscribers, and (6) verification of the devices.

We here distinguish two main phases, first the individual key construction and later the group key derivation. These phases are demonstrated in Figure 2 and Figure 3, respectively.

#### 4.2.1. Individual Key

The devices then start the ECQV protocol to derive their key pair. We slightly modify the protocol by sending an additional random EC point $G_{i}=g_{i}G$ to the CA in the certificate request. As a consequence, each device *i* sends $R_{i},ID_{i},G_{i}$ to the broker, who further forwards it to the CA.

Next, the CA aggregates $G_{i}$ also in the auxiliary data derivation $a_{i}$ and, thus, the final definition of the public key of the device becomes $Q_{i}=d_{i}G=H(ID_{i}∥C_{i}∥G_{i})C_{i}+Q_{CA}$. Similar as in the ECQV protocol, the CA sends the auxiliary data and certificate to the broker, who further forwards it to the device. Next, based on this information, the device is able to compute its private key and to verify the correctness of it.

See Figure 2 for a schematic overview of the communication messages between the node, broker and CA.

#### 4.2.2. Group Key

We distinguish six main steps in this phase.

1. Group key request of client.
  The device sends $d_{i}+g_{i}$ securely to the CA (via the broker) as response on the individual key construction, by encrypting it with the key $K=d_{i}Q_{CA}$. This allows the CA to verify the authenticity by checking if $\left(d_{i}+g_{i}\right)G=Q_{i}+G_{i}$ as only the device is capable to derive this value.
2. Group key request of client, forwarded by broker to CA.
  The broker collects all the info received by the devices and forwards it to the CA.
3. Construction of the group key related info by CA.
  Denote the identity of the group with $ID_{g}$, containing the group of publishers with identities $\left\{ID_{P1},\ldots ,ID_{Pn}\right\}$ and corresponding group of subscribers with identities $\left\{ID_{S1},\ldots ,ID_{Sn}\right\}$. For both groups, a secret key pair for the publishers $\left(d_{P},Q_{P}\right)$ and for the subscribers $\left(d_{S},Q_{S}\right)$ is derived. To this end, the CA first defines a group public key $Q_{CAg}$ by
  $$\begin{array}{ccc} Q_{CAg} & = & H(ID_{g}∥ID_{P1}∥\ldots ∥ID_{Pn}∥ID_{S1}∥\ldots ∥ID_{Sn}∥d_{CA})G\\ & = & d_{CAg}G. \end{array}$$
  The key pair $\left(d_{P},Q_{P}\right)$ of the publishers is then defined by
  (1) $$\begin{array}{cc} Q_{P}= & \sum_{i=1}^{n}Q_{pi}+\sum_{i=1}^{n}G_{pi}+Q_{CAg}\\ d_{P}= & \sum_{i=1}^{n}\left(d_{pi}+g_{pi}\right)+d_{CAg} \end{array}$$
  Similar for the key pair $\left(d_{S},Q_{S}\right)$ of the subscribers holds that
  (2) $$\begin{array}{cc} Q_{S}= & \sum_{i=1}^{n}Q_{si}+\sum_{i=1}^{n}G_{si}+Q_{CAg}\\ d_{S}= & \sum_{i=1}^{n}\left(d_{si}+g_{si}\right)+d_{CAg} \end{array}$$
4. Response of CA to broker.
  Consequently, the group info with group identity $ID_{G}$ contains the information
  $$\begin{array}{ccc} \{ & & (Q_{CAg},Q_{P},Q_{S},\\ & & \left(ID_{p1},C_{p1},G_{p1},Q_{p1}\right),\ldots ,\left(ID_{pn},C_{pn},G_{pn},Q_{pn}\right),\\ & & \left(ID_{s1},C_{s1},G_{s1},Q_{s1}\right),\ldots ,\left(ID_{sn},C_{sn},G_{sn},Q_{sn}\right)\;\;\;\;\}. \end{array}$$
  In addition, the CA also computes for each of the individual publishers and subscribers the encryption of $d_{P}$ and $d_{S}$, respectively, by means of the ECIES algorithm in which a random point R=rG is created in order to derive the common secret key $K=rQ_{i}$, where $Q_{i}$ is the public key of the publisher or subscriber.
  Both the group key information and the individual information is sent to the broker.
5. Response of broker to both publishers and subscribers.
  Upon receiving this information, the broker first checks the validity of the group key information of both the individual public keys (by the ECQV mechanism) and the group keys (by construction with $Q_{CAg}$). In the first two communication modes, these data should be publicly available and stored at the broker side. In the last mode, option 3 with anonymity, the broker only stores $\{\left(Q_{CAg},Q_{P},Q_{S})\right\}$.
  The broker then forwards to the publishers of the group
  $$\begin{array}{c} Q_{P},\left(ID_{s1},C_{s1},G_{s1},Q_{s1}\right),\ldots ,\left(ID_{sn},C_{sn},G_{sn},Q_{sn}\right), \end{array}$$
  and to the subscribers of the group
  $$\begin{array}{c} Q_{S},\left(ID_{p1},C_{p1},G_{p1},Q_{p1}\right),\ldots ,\left(ID_{pn},C_{pn},G_{pn},Q_{pn}\right). \end{array}$$
  Note that the broker is not able to verify the individual information as it does not know the private keys of the devices.
6. Verification of the devices.
  Based on the received info of the broker and the construction (Equations (1) and (2)), the publishers can validate the private group key $d_{S}$ and the subscribers the private group key $d_{P}$.

### 4.3. Secure Communication

We focus here on the communication of the message *M* by the publishers to the subscribers. Similar explanation holds for the communication from subscribers to publishers in case of a query. Denote the timestamp by *T*. We distinguish the three options as mentioned above.

- Option 1: Authentication and non-repudiation.
  Here, the publisher with identity $ID_{i}$ and key pair $\left(d_{i},Q_{i}\right)$ sends the message
  $$\begin{array}{ccc} M_{1} & = & \{ID_{G},ID_{i},T,M,V,s\}, \end{array}$$
  where V,s corresponds with the Schnorr signature and thus V=vG with a randomly chosen value *v* and $s=v-H(M∥T∥ID_{G})d_{i}$.
  Upon arrival of this message at the broker side, the broker can check the validity by verifying the signature. If correct, the broker further forwards the message to the subscribers of the group.
  In a similar way as the broker, the subscribers of the group can verify the authenticity of the message.
- Option 2: Authentication, non-repudiation, and confidentiality. In this case, the publisher sends the message
  $$\begin{array}{ccc} M_{2} & = & \{ID_{G},ID_{i},T,E_{K}\left(M\right),V,s\}, \end{array}$$
  with $K=H(d_{i}Q_{P}∥T)$. The parameters V,s are referring to the Schnorr signature and, thus, V=vG with a randomly chosen value *v* and $s=v-H(E_{K}\left(M\right)∥T∥ID_{G})d_{i}$.
  After receiving this message, the broker is able to verify the authentication by checking the signature. If correct, the broker further forwards the message to the subscribers of the group.
  The subscribers, who are in the possession of the private group key $d_{P}$ are able to decrypt the message and also to verify the individual authenticity of the message.
- Option 3: Authentication, confidentiality, non-repudiation, and anonymity.
  For the full option, the publishers send
  $$\begin{array}{ccc} M_{3} & = & \{ID_{G},T,Z,E_{K}\left(M,ID_{1},s_{1}\right),s_{2}\}, \end{array}$$
  with $K=H(zQ_{P}∥T)$ and Z=zG refers to the random value of the signature, while the two signatures $s_{1},s_{2}$ are defined as $s_{1}=z-H(E_{K}\left(M,ID_{1},s_{1}\right)∥T∥ID_{G})d_{i}$ and $s_{2}=z-H(E_{K}\left(M,ID_{1},s_{1}\right)∥T∥ID_{G})d_{S}$.
  The broker can again in the same way validate the authentication of the message at group level, while the subscribers are able to decrypt the message and to verify the authenticity.

### 4.4. Key Update Phase

In order to guarantee both backward and forward security, each time a new member enters or an old member leaves the publishing or subscribing group, the group public keys $Q_{CAg},Q_{P},Q_{S}$ and corresponding private keys $d_{CAg},d_{P},d_{S}$ need to be refreshed. Consider the following situation in which the publishing member $ID_{p1}$ is leaving the group. In this case, the CA defines the new public group key of the publishers $Q_{CAg}^{*}=H(ID_{g}∥ID_{P2}∥\ldots ∥ID_{Pn}∥ID_{S1}∥\ldots ∥ID_{Sn}∥d_{CA})G=d_{CAg}^{*}G$. As a consequence, the new $Q_{P}^{*}$ becomes $Q_{P}^{*}=Q_{P}-Q_{1}-Q_{CAg}+Q_{CAg}^{*}$ and the new $d_{P}^{*}=d_{P}-d_{1}-d_{CAg}+d_{CAg}^{*}$.

Additionally, $d_{S}$ and $Q_{S}$ should be updated accordingly by $d_{S}^{*}=d_{s}-d_{CAg}+d_{CAg}^{*}$ and $Q_{S}^{*}=Q_{s}-Q_{CAg}+Q_{CAg}^{*}$.

For the update process, the CA sends the new list of participants and corresponding security parameters, together with the new group keys $Q_{CAg}^{*},Q_{P}^{*},Q_{S}^{*}$ to the broker. The group of subscribers is able to securely receive $d_{P}^{*}$ by means of a broadcast communication using the previous $d_{P}$. However, for the publishers, the parameter $d_{S}^{*}$ should be individually sent to each of the members as the usage of $d_{S}$ will prevent backward security. Note that the devices do not need to update their individual key.

The process is similar for devices entering the group of publishers or for devices entering or leaving the group of subscribers.

## 5. Security Analysis

The security of the proposed protocol heavily relies on the security strength of well proven algorithms ECIES, ECQV, and Schnorr signature. We will explain more in depth the consequences for the construction of the key material and for the three different communication modes.

### 5.1. Construction of Key Material

First of all, due to the usage of the ECQV protocol, all nodes uniquely know their private key $d_{i}$ and their share of the group key $d_{i}+g_{i}$. Note that $d_{i}+g_{i}$ is also communicated to the CA. However, this information is still not sufficient to derive the unique private key $d_{i}$ in order to potentially impersonate one of the nodes. Moreover, the CA is considered to be fully trusted.

Thanks to the ECIES, the nodes also uniquely receive from the CA the private key for subscription of publishing $d_{S},d_{P}$, respectively.

### 5.2. Communication Modes

Here, there are the three modes to be discussed.

- Option 1: Thanks to the usage of the Schnorr signature and the construction of the key material, authentication, and non-repudiation are established.
- Option 2: Similar as in option 1, authentication and non-repudiation are established. In addition, due to the usage of the ECIES algorithm, also confidentiality is obtained.
- Option 3: The main difference with option 2 is that the identity of the sender is included in the encrypted message in order to obtain anonymity. As a consequence, the signature linked to that identity is also added to the encrypted message. An additional signature is included in order to allow the verification at group level by the broker. As a result, anonymity and unlinkablity are obtained as well.

## 6. Performance Analysis

In the proposed security model we assume the existence of a known CA server that will handle the security operations described along the paper. The public key of the CA server has to be pre-loaded in each end node to allow them to verify the origin of the certificate on certificate reception.

In the implementation of this security scheme, the broker is responsible of all the security operations to generate the certificate and the group keys without relying on an external CA server. This is, of course, not a safe situation, a malicious broker can store the group keys and generate the symmetric keys to decrypt all the messages it receives. However, it is sufficient to conduct an analysis of the interaction between the broker and the client nodes. As we can see in Figure 1, the broker just acts as a relay between the publisher and subscriber nodes and the CA server for the security operations. We will then consider them here both as the same entity.

### 6.1. Our Testbed

**Figure 4:**
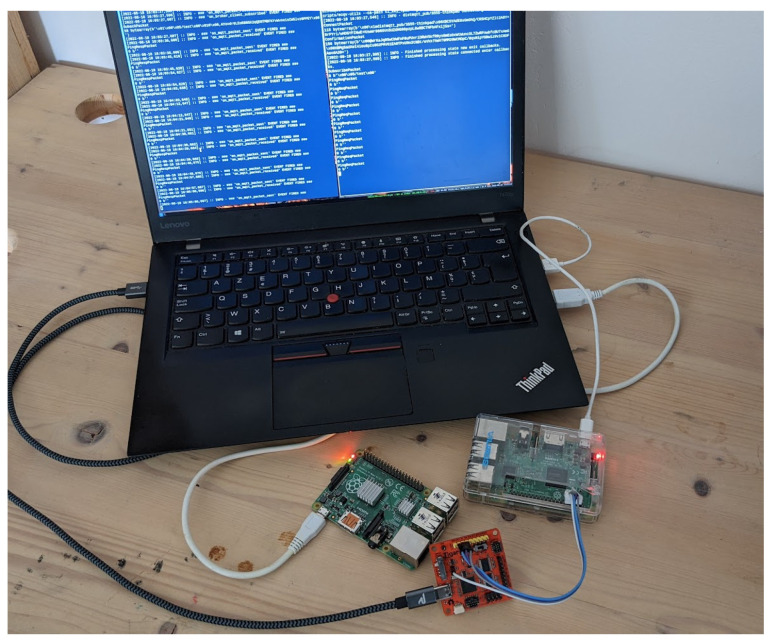
Testbed composed of a Raspberry PI 3B acting as the publisher connected to a laptop via a UART adapter that host the MQTT broker, a Raspberry Pi B+ acting as the subscriber.

The security scheme was implemented (https://github.com/tperale/distmqtt, accessed on 22 September 2022) to run on the Raspberry PI platform. The testbed consisted of a Raspberry PI 3B+ running a MQTT client and acting as a publisher connected via Wi-Fi to a laptop running the MQTT broker and a Raspberry PI B+ running a MQTT subscriber (shown in Figure 4. The DistMQTT implementation for MQTT was used since it was written in Python. All the measurements were taken on the publisher side on the Raspberry PI 3B+ and were monitored on the laptop using a UART adapter.

### 6.2. Overhead Analysis

The presented security scheme and its security keys exchanged between the nodes and the broker can be directly added to the original MQTT connection packet exchange. Section 4.2.2 described four steps to establish a secure communication between the publisher and subscriber. Similarly to [2], these steps can be directly embedded to the original MQTT packets by extending the payload of these packets. As a result, the number of packets sent between a node and the broker to make a connection is similar as for insecure MQTT, but some additional overhead is added in the payload.

1. Connection *(+certificate request)*. In the *connection* phase, the *connect* (see Table 1) packet is growing by 122 bytes to include the public key and the random number *g* of the pub/sub node.
2. Acknowledgement *(+certificate reception and validation)*. The *connack* packet (see Table 2) that acknowledges this connection now includes the ECQV certificate and the random number *r* needed for the certificate reception and thus adds a total of 98 bytes to this packet (see Figure 2).
3. Topic subscription *(+verification number sending)*. For the *verification* phase the *subscriptions* packet (see Table 3) must include the encrypted verification number which adds 48 bytes to the size of the packet.
4. Acknowledgement *(+group key reception)*. The *suback* packet (see Table 4) contains an encrypted public/private key pair which adds 98 bytes (see Figure 3).

To summarize, the whole scheme with these four phases adds a total of 366 bytes of communication overhead for the key initialization.

**Figure 5:**
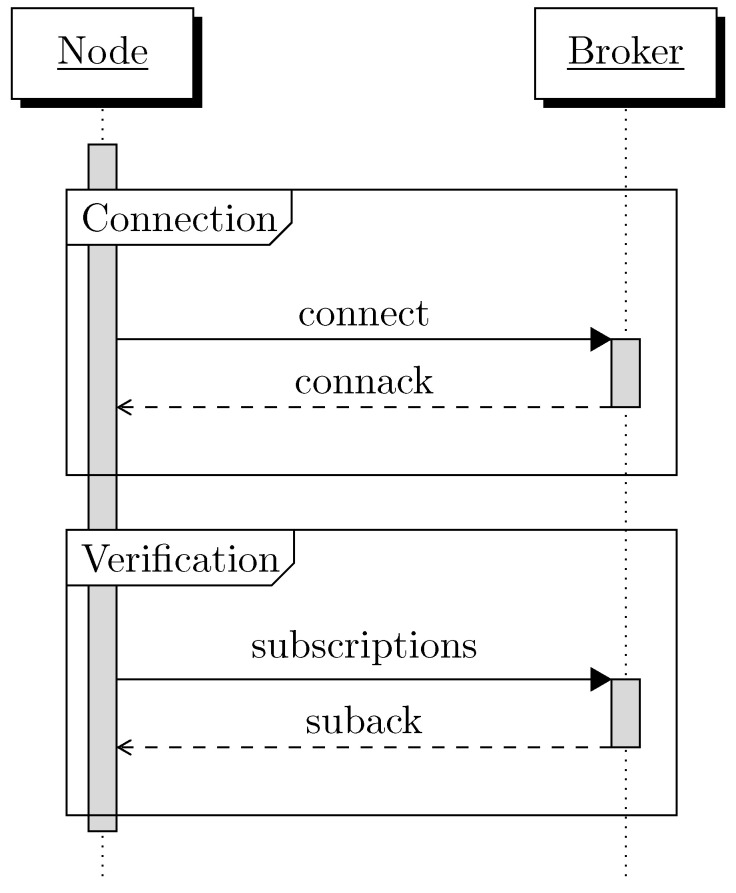
Connection procedure and security overhead in MQTTS. First, a secure connection with the broker is established and next the MQTT connection can be performed. Details on the connection and verification steps can be found in Figure 2 and Figure 3; the composition of the packets in Table 1, Table 2, Table 3 and Table 4.

In comparison, MQTTS requires the connecting node to establish a secure connection with the broker before performing the MQTT connection. Those steps are summarized in Figure 5 and were measured on algorithms using the secp256k1 curves.

**Figure 6:**
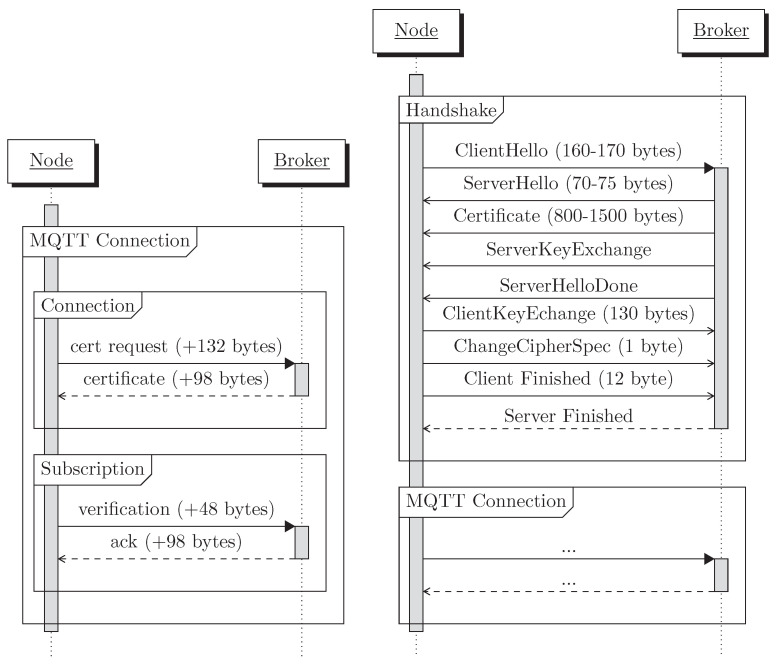
Comparison of the size overhead of our security scheme compared to a TLS 1.2 handshake. Details on TLS packets are shown in Table 5.

If we compare it to the values given in [2] (see Table 5, [2]) to establish a new TLS connection, our proposed scheme is highly efficient since a new TLS connection introduces an overhead of 1789 bytes, considering the use of only one certificate of 1500 bytes (four certificates are usually used [1]). In Figure 6, a visual representation of the message flows in our novel security scheme compared to the classical TLS handshake is presented. The figure highlights the difference in the number of packets exchanged between a node and the broker, depending on the security scheme. Our novel security scheme directly integrates the classical MQTT connection and does not increase the number of packets exchanged, contrary to TLS that requires to perform the handshake prior to the MQTT connection. Moreover, thanks to the usage of the implicit certificates, compared to the X.509 certificates in TLS, the messages are also much shorter. As a result, our protocol entails almost 80% less communication overhead, as compared to MQTTS.

### 6.3. Computational Cost

The design of this security scheme externalizes the computational complexity to the CA server. The nodes participating in the MQTT network are constrained devices that cannot handle expensive computations. There are two expensive operations performed on the client nodes: the certificate reception and the verification computation.

The reception of the ECQV certificate requires a hash operation (H), an elliptic curve point addition (PA), and a point multiplication (PM) to extract the private key of the certificate. The second operation is to generate the secret key to transmit the confirmation number to the broker of the node. This operation requires a point multiplication and an AES encryption. The overhead of those two functions were measured on the MQTT client running on a Raspberry Pi 3B. The overhead is summarized in Table 6. The presented security scheme tested on Raspberry Pi 3B running in a MQTT network adds an overhead mean is between 49.3 ms and 49.7 ms with 95% confidence interval.

**Figure 7:**
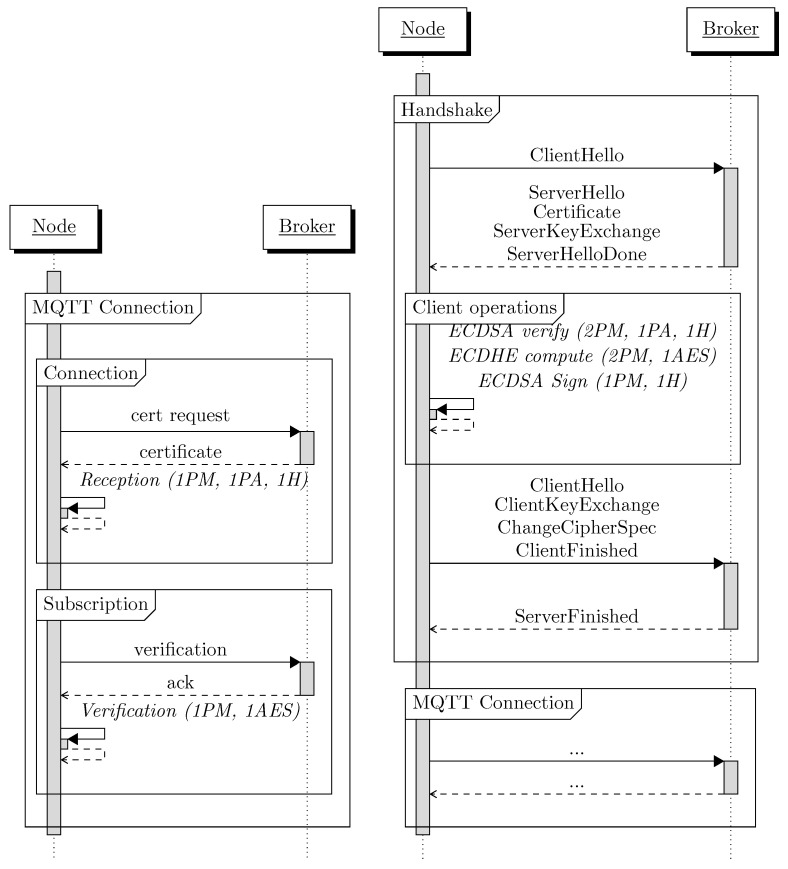
Comparison of the computational overhead of our security scheme compared to a TLS 1.2 handshake based on ECDH_ECDSA.

This security scheme can be compared to the performance overhead of the TLS handshake but this metric depends on the type of certificate exchanged (see [26]). In Figure 7, the performance of the TLS handshake based on ECDH_ECDSA certificate is compared to our security scheme. We only measure the computationally intensive part of handshake where the symmetric key is computed (the client operation part of Figure 7). This part is made of three different computationally expensive operations: the verification, the computation of ECDHE key and its signature, but we will measure them as a whole because they are completed inside the same step of the TLS handshake. The number for MQTTS of Table 6 is measured during a handshake initiated from a Raspberry PI 3B using a secp256r1 curve and takes a total of 144 ms.

We can compare the number of the two security scheme from Table 6. The computational overhead of our security scheme is smaller than the one of the traditional TLS handshake. To be more specific, TLS demands almost 3 times the computational overhead of our security scheme. For this we need to add the delay of the packet exchanged by the handshake. However, as this delay is dependant on the network connection, we do not go into details.

### 6.4. Scalability

A new set of topic group keys is generated each time a node joins a topic. The equations to generate the group keys are defined by Equations (1) and (2). The CA server only requires from the participating nodes to identify each topic to re-generate the pub/sub group keys. The identity is required to generate the group key pair.

The set of new topic pub/sub group keys is generated by just adding the new verification number of the joining node to the previous pub/sub group key and re-generating the group private key. Verification numbers do not need to be stored indefinitely by the CA server which keeps the storage overhead in the CA server per topic constant, regardless the number of participants.

## 7. Conclusions

This paper presents a flexible and lightweight MQTT security scheme developed for constrained devices. It overcomes the issue of a dishonest broker which has the capability to retrieve all messages sent to it. Our proposed scheme ensures the confidentiality and non-repudiation by sharing a different key pair for the publishers and subscribers of each topic. It relies on a trusted external certificate authority responsible for the generation of the security keys. The CA public key is known and pre-loaded on each client node to verify that the security keys were correctly generated by a trusted CA. The messages sent on each topic are signed and encrypted to allow the recipients to verify that they originate from a “publisher” node.

This novel security scheme reduces the communication overhead of TLS by approximately 80% (when comparing the amount of data exchanged) because the scheme does not require a handshake before performing an MQTT connection. The number of packets sent between the client and the broker which reduces the delay to establish a secure connection. Additionally, the number of cryptographic operations used in our scheme is much lower than the one used in traditional TLS and reduces the computational overhead of the added security by approximately 3 times.

Future work should improve the scalability of the security scheme to avoid the re-computation of the topic keys for each connection/disconnection. Additionally, MQTTS should be considered to port this security scheme to small devices. Currently, the architecture relies on a semi-trusted broker and a fully trusted CA. Note that for the key initialization, thanks to the ECQV mechanism, the CA can be still considered honest but curious. However, its strongest involvement is in Step 3, construction of the group key related info, where it defines the key pair for both the groups of the publishers and the subscribers and, thus, a completely trusted CA is needed. It is an open problem and part of future work to investigate how the scheme can be developed with also an honest and curious CA without significant performance cost.

## Figures and Tables

**Table 1:** MQTT modified connection packet.

Fixed Header (2 bytes)				
packet_type	remaining_length	flags		
Variable Header (4 bytes)				
proto_name	proto_level	flags		keep_alive
Payload (variable)				
client_id	pk (+66 bytes)	g (+66 bytes)		

**Table 2:** MQTT modified connack packet.

Fixed Header (2 bytes)			
packet_type		remaining_length	flags
Variable Header (2 bytes)			
session_parent		return_code	
Payload (98 bytes)			
cert (+32 bytes)		r (+66 bytes)	

**Table 3:** MQTT modified subscribe packet.

Fixed Header (2 bytes)					
packet_type		remaining_length		flags	
Variable Header (2 bytes)					
packet_id					
Payload (variable)					
topics			verification (+ 48 bytes)		

**Table 4:** MQTT modified suback packet.

Fixed Header (2 bytes)					
packet_type		remaining_length		flags	
Variable Header (2 bytes)					
packet_id					
Payload (variable)					
topics	return_codes	group_keys private (+32 bytes)		group_keys public (+66 bytes)	

**Table 5:** Message sizes of TLS.

Message	Size	Remarks
ClientHello	160–170 bytes	Depends on parameter such as cipher suites. Client Hello extensions and session resumption.
Session ID	132 bytes	
ServerHello	70–75 bytes	Varies with Server Hello extensions.
Certificate	800–1500 bytes	Depends on the certificate chain size and the number of certificates needed.
ClientKeyExchange	130 bytes	
ChangeCipherSpec	1 byte	
Finished	12 bytes	
TLS Record Header	5 bytes	
TLS Handshake Header	4 bytes	

**Table 6:** Comparison of computational overhead of the operations on a Raspberry PI 3B in our proposed scheme versus MQTTS.

Step (Proposed Scheme)	Operations	Time (ms)
Certificate reception	1 PM, 1 PA, 1 H	29
Verification	1 PM, 1 AES	20
**Step (MQTTS)**	**Operations**	**Total Time (ms)**
ECDSA Verification	2 PM, 1 PA, 1 H	
ECDHE	2 PM, 1 AES	144
ECDSA Signature	1 PM, 1 H	
